# Oral Microbiome Profile of the US Population

**DOI:** 10.1001/jamanetworkopen.2025.8283

**Published:** 2025-05-05

**Authors:** Anil K. Chaturvedi, Emily Vogtmann, Jianxin Shi, Yukiko Yano, Martin J. Blaser, Nicholas A. Bokulich, J. Gregory Caporaso, Maura L. Gillison, Barry I. Graubard, Xing Hua, Autumn G. Hullings, Lisa Kahle, Rob Knight, Shilan Li, Jody McLean, Vaishnavi Purandare, Yunhu Wan, Neal D. Freedman, Christian C. Abnet

**Affiliations:** 1 Division of Cancer Epidemiology and Genetics, National Cancer Institute, Bethesda, Maryland; 2 Center for Advanced Biotechnology and Medicine, Rutgers University, Piscataway, New Jersey; 3 Department of Health Sciences and Technology, ETH Zurich, Zurich, Switzerland; 4 Center for Applied Microbiome Science, Pathogen and Microbiome Institute, Northern Arizona University, Flagstaff; 5 Department of Thoracic and Head and Neck Medical Oncology, MD Anderson Cancer Center, Houston, Texas; 6 Information Management Services, Calverton, Maryland; 7 Center for Microbiome Innovation, University of California, San Diego, La Jolla; 8 Bloomberg School of Public Health, Johns Hopkins University, Baltimore, Maryland; 9 National Center for Health Statistics, Centers for Disease Control and Prevention, Hyattsville, Maryland; 10 Division of Cancer Control and Population Sciences, National Cancer Institute, Bethesda, Maryland

## Abstract

**Importance:**

The oral microbiome likely plays key roles in human health. Yet, population-representative characterizations are lacking.

**Objective:**

To characterize the composition, diversity, and correlates of the oral microbiome in US adults.

**Design, Setting, and Participants:**

This cross-sectional study analyzed data from the population-representative National Health and Nutrition Examination Survey (NHANES) from 2009 to 2012. Microbiome data were made publicly available in 2024. NHANES participants were aged 18 to 69 years and provided oral rinse samples in 1 of 2 consecutive NHANES cycles (2009-2010 and 2011-2012).

**Exposures:**

Demographic, socioeconomic, behavioral, anthropometric, metabolic, and clinical characteristics.

**Main Outcomes and Measures:**

Oral microbiome measures, characterized through 16S ribosomal RNA gene sequencing, included α diversity (observed amplicon sequence variants [ASVs], Faith phylogenetic diversity, Shannon-Weiner Index, and Simpson Index); β diversity (unweighted UniFrac, weighted UniFrac, and Bray-Curtis dissimilarity); and prevalence and relative abundance at phylum level through genus level. Analyses accounted for the NHANES complex sample design.

**Results:**

This study included 8237 US adults aged 18 to 69 years, representing 202 314 000 individuals (102 813 000 men [50.8%]; mean [SD] age, 42.3 [14.4] years; 9.3% self-reported as Mexican American, 12.1% as non-Hispanic Black, 64.7% as non-Hispanic White, 5.9% as other Hispanic, and 8.1% as other non-Hispanic individuals). The oral microbiome encompassed 37 bacterial phyla, 99 classes, 212 orders, 446 families, and 1219 genera. Five phyla (*Firmicutes, Actinobacteria, Bacteroidetes, Proteobacteria,* and *Fusobacteria*) and 6 genera (*Veillonella, Streptococcus, Prevotella 7, Rothia, Actinomyces,* and *Gemella*) were present in nearly all US adults (weighted prevalence, >99%). These genera were the most abundant, accounting for 65.7% of total abundance. Observed ASVs showed a quadratic pattern with age (peak at 30 years), were similar by sex, significantly lower among non-Hispanic White individuals, and increased with greater body mass index (BMI), alcohol use, and periodontal disease severity. All covariates together accounted for a modest proportion of oral microbiome variability as measured by β diversity: *R*^2^ = 8.7% (95% CI, 8.4%-9.1%) for unweighted UniFrac, *R*^2^ = 7.2% (95% CI, 6.6%-7.7%) for weighted UniFrac, and *R*^2^ = 6.3% (95% CI, 3.1%-6.7%) for Bray-Curtis matrices. By contrast, relative abundance of a few genera explained a high percentage of variability in β diversity for weighted UniFrac: *Aggregatibacter* (*R*^2^ = 22.4%; 95% CI, 22.1%-22.8%), *Lactococcus* (*R*^2^ = 21.6%; 95% CI, 20.9%-22.3%), and *Haemophilus* (*R*^2^ = 18.4%; 95% CI, 18.1%-18.8%). Prevalence and relative abundance of numerous genera were associated with age, race and ethnicity, smoking, BMI categories, alcohol use, and periodontal disease severity.

**Conclusions and Relevance:**

This cross-sectional study of the oral microbiome in US adults showed that a few genera were universally present and a different set of genera explained a high percentage of oral microbiome diversity across the population. This comprehensive characterization provides a contemporary reference standard for future studies.

## Key Points

**Question:**

What are the composition, diversity, and correlates of the oral microbiome in the US population?

**Findings:**

In this cross-sectional study of the oral microbiome data of 8237 adults in the population-representative National Health and Nutrition Examination Survey, 5 phyla and 6 genera were detectable in nearly all individuals. Although less than 9% of the variability in the oral microbial communities was explained by participant characteristics and behaviors, age, race and ethnicity, periodontal disease, and smoking were associated with the prevalence and relative abundance of genera.

**Meaning:**

This comprehensive characterization provides a contemporary reference standard of the oral microbiome of US adults for future studies.

## Teaser

This cross-sectional study examines the demographic, socioeconomic, behavioral, anthropometric, metabolic, and clinical characteristics of the oral microbiome of US adults.

## Introduction

The community of bacteria and other microbiota in the human oral cavity, the oral microbiome, is increasingly recognized to play a role in health and disease.^1,2,3^ The oral microbiome is involved in digestion and metabolism of nutrients (eg, dietary nitrates and carbohydrates) and carcinogens (eg, conversion of ethanol to acetaldehyde), blood pressure and metabolic homeostasis,^1,4,5^ and regulation of oral and systemic immunity and inflammation.^1,4^ Certain oral bacteria contribute to periodontitis and poor oral health,^6^ and alterations in the oral microbiome have been associated with several chronic diseases, including cancer (eg, upper aerodigestive and colorectal), diabetes, cardiovascular disease, and neurological disorders.^1,3,4,7,8,9,10,11^

Understanding the relevance of the oral microbiome in human health requires a population reference standard of the composition of the oral bacterial ecosystem and prevalence, relative abundance, and interrelationships of taxa, established through characterization of the oral microbiome in a representative population sample. Such a reference standard could serve as a critical comparator to explain the disease relevance of alterations in the oral microbiome. However, few population-representative characterizations exist in the literature.^12^ Most prior studies of the oral microbiome have been conducted in nonrepresentative groups or highly selective subgroups.^2,13,14,15^

Here, we address the lack of population-representative studies. Specifically, we aimed to characterize the composition; diversity; and demographic, socioeconomic, behavioral, anthropometric, metabolic, and clinical correlates of the oral microbiome in a representative sample of US adults.^16^

## Methods

### Study Population and Biospecimens

We obtained data from the National Health and Nutrition Examination Survey (NHANES), a complex, stratified, multistage cluster probability sample of the civilian, noninstitutionalized US population.^17,18^ NHANES includes a household interview component for collection of data on demographics, diet, tobacco use, and medical history as well as a mobile examination center (MEC) component for health, dental, anthropometric, and biochemical examinations and biospecimen collection.^17,18^ The NHANES protocol was approved by the National Center for Health Statistics (NCHS) Ethics Review Board. All NHANES participants provided written informed consent. This cross-sectional study is covered by the overall protocol. We followed the Strengthening the Reporting of Observational Studies in Epidemiology (STROBE) reporting guideline.

We conducted this study using oral rinse samples collected for oral human papillomavirus (HPV) evaluations in 2 consecutive NHANES cycles (2009-2010 and 2011-2012).^19^ Briefly, NHANES incorporated an oral HPV protocol in 4 cycles (2009-2016) for participants aged 14 to 69 years.^20,21^ Response rates for the MEC component were 68.5% in the 2009-2010 cycle and 69.5% in the 2011-2012 cycle.

### Laboratory Processing and Bioinformatics

NHANES participants provided a 10-mL oral rinse (Scope mouthwash or saline) sample, which were shipped to the Gillison laboratory (Ohio State University). DNA was extracted on the QIAsymphony SP instrument (QIAGEN) using the Virus/Bacteria Midi Kit and Pathogen Complex 800 program (QIAGEN).^19^

DNA sequencing methods are described in detail in the data documentation.^22^ Extracted DNA was shipped to the Knight laboratory (University of California, San Diego), and the V4 region of the 16S ribosomal RNA (rRNA) gene was amplified using polymerase chain reaction and sequenced using paired-end sequencing of 125 base pair^23^ (HiSeq 2500; Illumina, Inc). However, only data from forward sequencing runs were used for analyses due to a lack of overlapping reads.

During the study period, the policy of the NCHS, which oversees NHANES, did not allow targeting human DNA for sequencing due to participant confidentiality concerns, which precluded metagenomic sequencing, so we targeted only eubacteria. Consequently, our findings lack species-level resolution and imputed bacterial functionality. Additionally, there are geographic bacterial niches within the oral cavity^1,2,24,25^; thus, our results from oral rinses need to be interpreted as the generalized or sentinel microbiome profile of the biofilms within the oral cavity.

Quality control analyses showed acceptable test-retest reproducibility.^22^ There was no evidence for plate or batch effects (eFigures 1 and 2 in Supplement 1).

The FASTQ files were demultiplexed using the QIIME 1 open-source software.^26^ The forward reads from each sequencing run were processed using the DADA2 method, and amplicon sequence variants (ASVs) were estimated.^27,28^ The phylogenetic tree was generated using QIIME 2,^29^ and taxonomy was assigned using the SILVA, version 123 database.^30^ For primary analyses, we created an ASV table that filtered out a nonbacterial ASV (SV1032, mapping to a mitochondrial pseudogene) that was identified in data quality checks.^22^

The resulting ASV tables were rarefied 10 times at 10 000 reads per sample and averaged for calculation of α diversity metrics. We calculated 4 α diversity metrics using ASV tables: number of observed ASVs, a measure of richness of samples; the Faith phylogenetic diversity, a phylogeny-weighted richness; the Shannon-Weiner Index, a measure of evenness of samples; and the Simpson Index, a measure of both richness and evenness of samples.^22^ Additionally, we calculated 3 β diversity distance matrices for n × n comparisons across participants as measures of differences in the bacterial community after rarefaction to 10 000 reads: unweighted UniFrac, which considers presence or absence of bacterial ASVs to estimate phylogeny-weighted distance between participants; weighted UniFrac, which considers relative abundance of bacterial ASVs to estimate phylogeny-weighted distance between participants; and Bray-Curtis dissimilarity, which considers relative abundance of bacterial ASVs without phylogenetic weighting.^22^

Taxonomic analyses were based on nonrarefied data but were restricted to samples with at least 10 000 reads. Bacterial taxonomy was characterized across phylum, class, order, family, and genus levels, with data expressed as prevalence (presence or absence) and relative abundance (ratio of the number of each taxon-specific sequences and sum of all taxon-specific sequences in an individual). We present results primarily at the phylum and genus levels; taxonomic identifiers and prevalence estimates at all levels are presented in eTables 1 to 6 in Supplement 2.

DNA sequencing and bioinformatics were completed in 2019. Due to COVID-19–induced delays, data quality control occurred from 2020 to 2022. The α and β diversity results were made publicly available in 2023, and taxonomic data were made publicly available in 2024.^22^

### Statistical Analysis

All analyses accounted for the NHANES complex sampling through the use of the cycle-specific NHANES sample weights (examination weights adjusted for nonresponse) and the strata and multistage-clustered sampling design. We created poststratification adjustments to the cycle-specific examination weights to account for nonavailability of oral microbiome results (18.4%) from MEC participants aged 18 to 69 years (eAppendix 1 in Supplement 1). Specifically, MEC weights were poststratified to the age by sex and by race and ethnicity cycle-specific distribution of the US population. Poststratified weights were divided by 2 given the use of 2 NHANES cycles.

We described the structure and diversity of the oral microbiome through descriptive statistics. Results were presented as percentages, means, medians, and quantiles, as appropriate. Taxa by taxa relative abundance correlations were calculated using sample-weighted Pearson correlation coefficients (eTables 7-11 in Supplement 2). Population totals were estimated using poststratified weights.

Analyses considered demographic and socioeconomic factors (age [modeled using restricted 5-knot cubic regression splines], sex, race and ethnicity, educational level, marital status, and income to poverty ratio), anthropometrics (measured body mass index [BMI]; categorized as underweight, normal weight, overweight, obesity, and severe obesity), risk behaviors (smoking and alcohol use), medical conditions (diabetes and hypertension), oral health (periodontal disease, tooth count, and edentulism), and use of prescription medications within the past 30 days (antibiotics, antilipidemics, respiratory inhalants, and gastroesophageal reflux drugs). Race and ethnicity were self-reported as Mexican American, non-Hispanic Black (hereafter Black), non-Hispanic White (hereafter White), other Hispanic, and other non-Hispanic (including but not limited to Asian, Native American, Pacific Islander, and multiracial). These variables were chosen a priori for adjustment in statistical models (variable definitions provided in eAppendix 2 in Supplement 1).

Correlates of the 4 α diversity metrics were evaluated using multiple linear regression, with each metric as the dependent variable. Results from these models were expressed as predictive margins, analogous to adjusted means.^31^ For the 3 β diversity matrices, we estimated sample-weighted principal coordinate analysis (PCoA) vectors and used the first 100 PCoAs (scree plots in eFigure 3 in Supplement 1) as outcomes in linear regression models.

We applied the Fast Adonis algorithm^32^ to estimate partial- and full-model explained variability in the β diversity matrices from each variable and combinations of variables using the analysis of variance principle while accounting for the complex design. Similarly, we estimated the contribution of each genus to variability in the β diversity matrices, unadjusted for covariates or correlations across genera.^32^

Our analyses of covariate associations examined genus-level associations with prevalence of genera using binary logistic regression, with results presented as adjusted odds ratios (AORs). Associations with relative abundance were examined using Poisson regression, with genus-level sequence counts as the outcomes and the total sequence counts as the offset; results were presented as adjusted risk ratios (ARRs). We note that microbiome relative abundance data seldom follow a Poisson distribution and show zero inflation, which results in variance inflation. It is, however, difficult to implement zero-inflated regression (eg, zero-inflated negative binomial or Poisson regression) while accounting for the NHANES design because of recognized issues with model convergence. Thus, we used complex survey design–based Poisson regression, which provides robust variances that account for extra Poisson variability. Logistic and Poisson regression models were adjusted for the covariates except respiratory inhalant drugs (excluded due to sparse sample sizes).

Associations with α and β diversity used 2-sided Wald *P* < .05 for statistical significance. Analyses of the genus-level prevalence and relative abundance were restricted to genera with at least 1% prevalence (n = 229 genera) and used a Bonferroni-corrected Wald *P* < .0002 for statistical significance. Analyses were conducted in SAS-callable SUDAAN, version 10.4.1 (RTI International) and R, version 4.2.0 (R Project for Statistical Computing).

## Results

**Figure 1.**
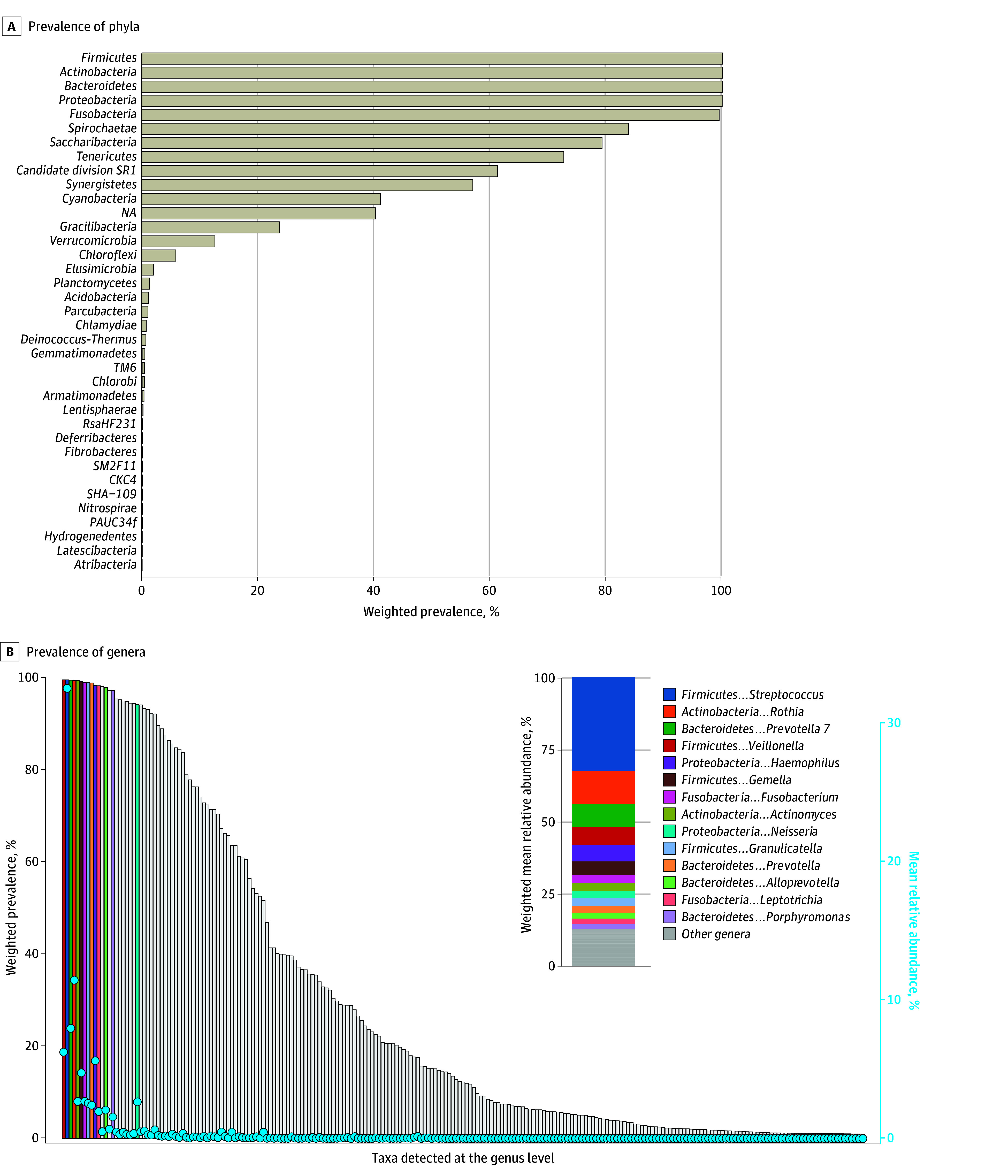
Prevalence of Phyla and Genera in the Oral Microbiome of Adults in the 2009 to 2012 National Health and Nutrition Examination Surveys Circles represent weighted mean relative abundance. The inset in panel B shows the proportionate weighted mean relative abundance for genera with more than 1% mean relative abundance and for the remaining genera. Prevalence estimates of taxa at the phylum and genus levels are shown in eTables 2 and 6, respectively, in Supplement 2. NA indicates not applicable.

Among 8237 US adults aged 18 to 69 years (representing 202 314 000 individuals; 99 501 000 women [49.2%], 102 813 000 men [50.8%]; mean [SD] age, 42.3 [14.4] years; 12.1% self-reported as Black, 9.3% as Mexican American, 64.7% as White, 5.9% as other Hispanic, and 8.1% as other non-Hispanic individuals), the oral microbiome encompassed 37 bacterial phyla, 99 classes, 212 orders, 446 families, and 1219 genera. Five phyla (*Firmicutes, Actinobacteria, Bacteroidetes, Proteobacteria,* and *Fusobacteria*) and 6 genera (*Veillonella, Streptococcus, Prevotella 7, Rothia, Actinomyces,* and *Gemella*) were present in nearly all adults (weighted prevalence, >99%) (Figure 1). These genera also had the highest relative abundances (Figure 1B), collectively accounting for 65.7% of the genus-level total abundance: *Streptococcus* (32.6%), *Rothia* (11.5%), *Prevotella 7* (8.0%), *Veillonella* (6.3%), *Gemella* (4.8%), and *Actinomyces* (2.7%). At the population level, however, genus-level prevalence was only moderately correlated with its relative abundance (ρ = 0.35), as most prevalent genera had low relative abundances.

### α Diversity Associations

**Figure 2.**
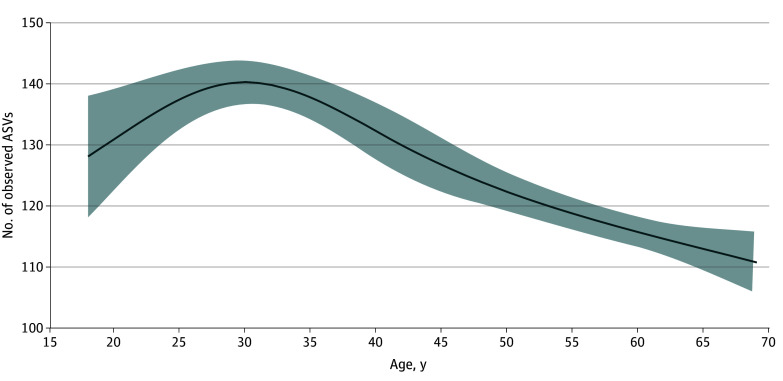
Adjusted Predictive Margins for the Number of Observed Amplicon Sequence Variants (ASVs) by Age in the Oral Microbiome of Adults in the 2009 to 2012 National Health and Nutrition Examination Surveys Age was modeled through 5-knot restricted cubic splines. Predictive margins (solid line) were estimated in survey design–adjusted linear regression models, with concomitant adjustment for sex, self-reported race and ethnicity, educational level, marital status, income to poverty ratio, body mass index, risk behaviors (smoking and alcohol use), medical conditions (diabetes and hypertension), oral health (periodontal disease, tooth count, and edentulism), and use of prescription medications within the past 30 days (antibiotics, antilipidemics, respiratory inhalants, and for gastroesophageal reflux disease [GERD]). eAppendix 2 in Supplement 1 provides variable definitions. The shaded area represents the 95% CIs.

The estimated mean oral microbiome α diversity in adults was 128 (95% CI, 125-130) for observed ASVs, 14.5 (95% CI, 14.3-14.7) for the Faith phylogenetic diversity, 4.58 (95% CI, 4.55-6.61) for the Shannon-Weiner Index, and 0.90 (95% CI, 0.89-0.90) for the Simpson Index. Across ages, the number of observed ASVs showed a nonlinear quadratic pattern, with an increase from age 18 to 30 years, peak at age 30 years, and subsequent decline after age 30 years (Figure 2). The number of observed ASVs was similar for men and women (126 and 129). Compared with White individuals (124 [95% CI, 121-127] ASVs), observed ASVs were significantly higher for individuals of other race and ethnicity groups: Black (134; 95% CI, 131-138); Mexican American (137; 95% CI, 133-140); other Hispanic (132; 95% CI, 129-136); and other non-Hispanic (131; 95% CI, 127-135), including Asian (127), a group first oversampled in the 2011 to 2012 NHANES (eFigure 4 in Supplement 1). Observed ASVs decreased with higher educational level and increased with greater BMI, alcohol use (among ever drinkers), and severity of periodontal disease (eFigure 4 in Supplement 1). The number of observed ASVs was substantially lower in edentulous individuals compared with the population mean (75 [95% CI, 68-83] vs 128 [95% CI, 125-130]). Use in the past 30 days of prescription antibiotics, antilipidemic medications, and hypertension and gastroesophageal reflux drugs was associated with lower observed ASVs, while use of inhaled respiratory drugs did not alter this metric (eFigure 4 in Supplement 1). Similar associations were observed for the other α diversity metrics (eFigure 4 in Supplement 1).

### β Diversity Associations

**Figure 3.**
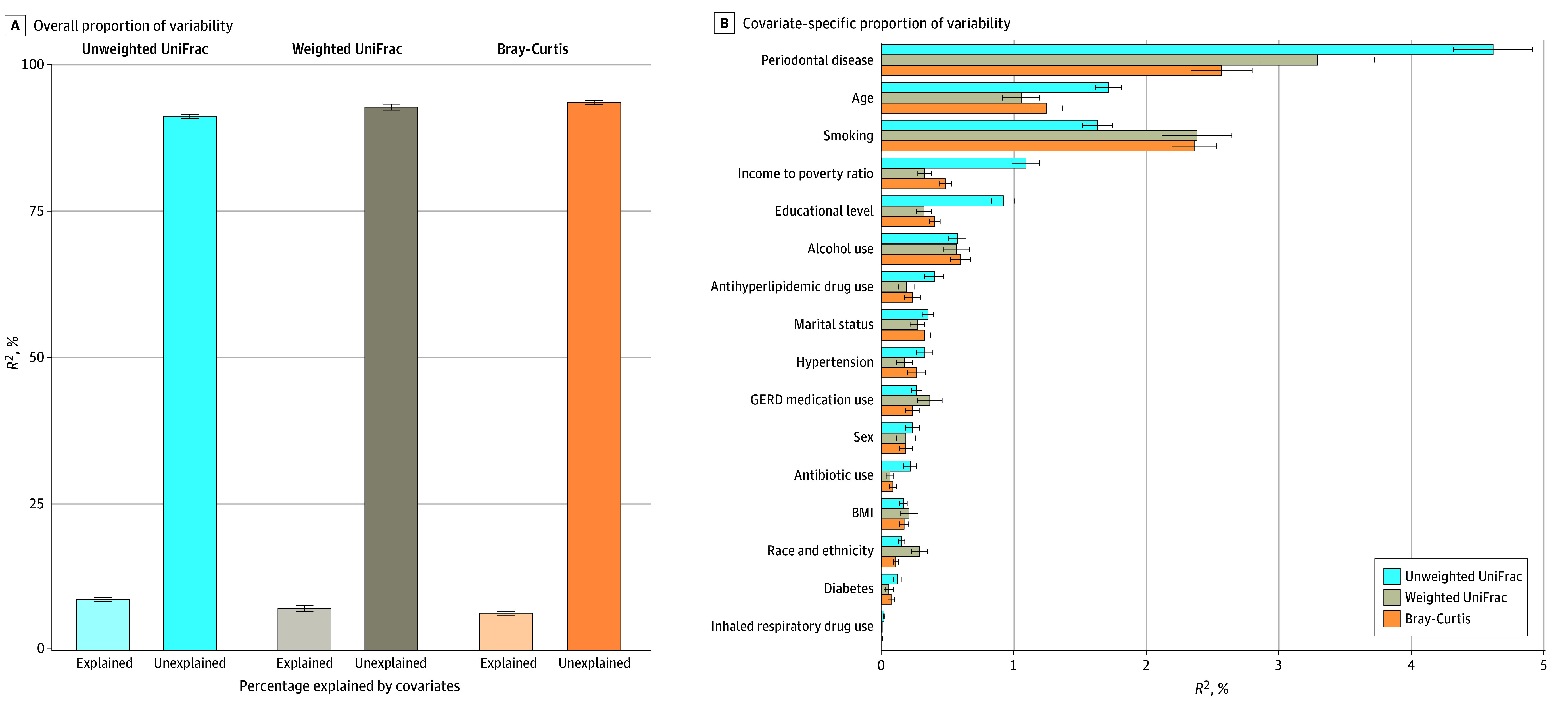
Overall and Covariate-Specific Proportion of Variability in β Diversity Matrices in the Oral Microbiome of Adults in the 2009 to 2012 National Health and Nutrition Examination Surveys The estimates were based on models that incorporated concomitant adjustment for age (modeled as 5-knot restricted cubic splines), sex, self-reported race and ethnicity, educational level, marital status, income to poverty ratio, body mass index (BMI), risk behaviors (smoking and alcohol use), medical conditions (diabetes and hypertension), oral health (periodontal disease, tooth count, and edentulism), and use of prescription medications within the past 30 days (antibiotics, antilipidemics, respiratory inhalants, and for gastroesophageal reflux disease). eAppendix 2 in Supplement 1 provides variable definitions. The Fast Adonis algorithm was used for estimation. Error bars represent 95% CIs.

A modest percentage (<9%) of the overall variability in β diversity was explained by the demographic, socioeconomic, behavioral, and health covariates: *R*^2^ = 8.7% (95% CI, 8.4%-9.1%) for unweighted UniFrac, *R*^2^ = 7.2% (95% CI, 6.6%-7.7%) for weighted UniFrac, and *R*^2^ = 6.3% (95% CI, 3.1%-6.7%) for Bray-Curtis matrices (Figure 3). Dominant factors in weighted UniFrac analyses for β diversity variability were periodontal disease (*R*^2^ = 3.3%; 95% CI, 2.9%-3.7%), age (*R*^2^ = 1.1%; 95% CI, 0.9%-1.2%), and smoking (*R*^2^ = 2.4%; 95% CI, 2.1%-2.7%). These covariates and antibiotic use were also associated with the first PCoA axis across the 3 β diversity matrices (eTables 12-14 in Supplement 2).

By contrast, a few genera explained a surprisingly high percentage of variability in the β diversity matrices, in particular weighted UniFrac from relative abundance of 3 genera: *Aggregatibacter* (*R*^2^ = 22.4%; 95% CI, 22.1%-22.8%), *Lactococcus* (*R*^2^ = 21.6%; 95% CI, 20.9%-22.3%), and *Haemophilus* (*R*^2^ = 18.4%; 95% CI, 18.1%-18.8%) (eFigure 5 in Supplement 1). These genera had marked variation in mean prevalence and relative abundance compared with each other: 67.3% (95% CI, 65.8%-68.8%) and 0.47% (95% CI, 0.44%-0.45%) for *Aggregatibacter*, 5.5% (95% CI, 4.9%-6.1%) and 0.007% (95% CI, 0.001%-0.01%) for *Lactococcus*, and 98.3% (95% CI, 97.9%-98.7%) and 5.6% (95% CI, 5.4%-5.8%) for *Haemophilus*. These results underscore these genera’s roles in β diversity, particularly in weighted UniFrac, regardless of prevalence or relative abundance.

### Genus-Level Associations

The prevalence and/or relative abundance of genera with major contributions to variability in weighted UniFrac (*Aggregatibacter* and *Lactococcus*) also had associations with several covariates. For example, the prevalence of *Aggregatibacter* was associated with race and ethnicity (Black vs White: AOR, 1.74 [95% CI, 1.37-2.21]; Mexican American vs White: AOR, 1.90 [95% CI, 1.55-2.32]; other Hispanic vs White: AOR, 1.82 [95% CI, 1.49-2.22]; and other races and ethnicities vs White: AOR, 1.74 [95% CI, 1.37-2.21]), smoking (former vs never smoker: AOR, 0.88 [95% CI, 0.74-1.04]; current vs never smoker: AOR, 0.69 [95% CI, 0.60-0.80]), periodontal disease (mild vs none: AOR, 1.13 [95% CI, 0.78-1.64]; moderate vs none: AOR, 1.25 [95% CI, 1.01-1.53]; severe vs none: AOR, 1.63 [95% CI, 1.21-2.20]; ineligible for assessment vs none: AOR, 0.98 [95% CI, 0.65-1.47]; edentulous vs none: AOR, 0.18 [95% CI, 0.11-0.29]; missing assessment vs none: AOR, 0.95 [95% CI, 0.66-1.39]), and antibiotic use (AOR, 0.31; 95% CI, 0.23-0.41) (eTable 15 in Supplement 2). Relative abundance of *Lactococcus* was 4.8-times higher among antibiotic users than nonusers (ARR, 4.8; 95% CI, 2.8-8.1) and 3.2-times higher among individuals with severe obesity vs normal weight (ARR, 3.2; 95% CI, 1.3-8.3) (eTable 16 in Supplement 2).

**Figure 4.**
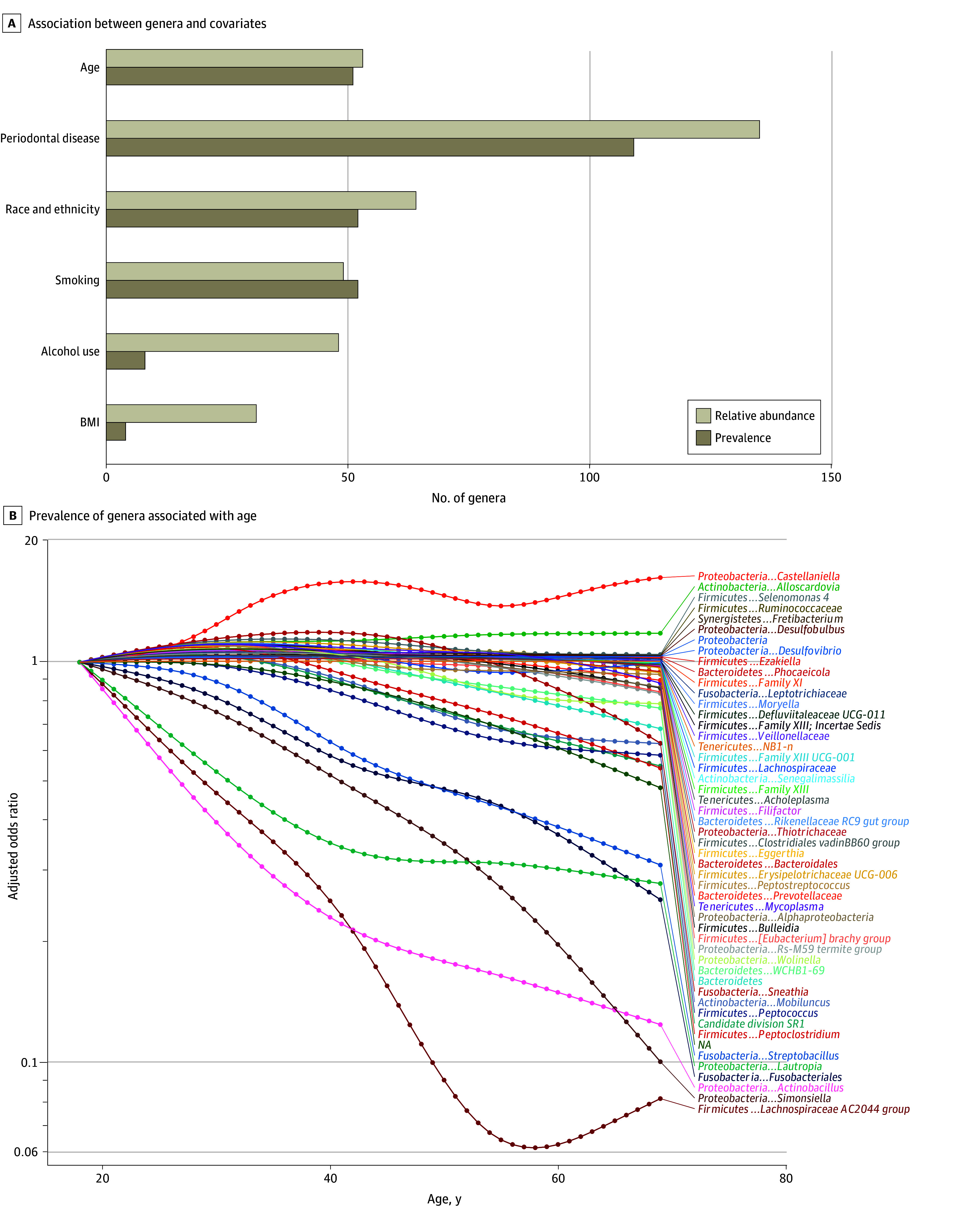
Covariates Associated With the Number of Genera and Adjusted Odds Ratio by Age for Prevalence of Genera in Adults in the 2009 to 2012 National Health and Nutrition Examination Surveys Associations with relative abundance were estimated in Poisson regression models. Associations with prevalence were estimated in binary logistic regression models. All models included concomitant adjustment for age (modeled as 5-knot restricted cubic splines), sex, self-reported race and ethnicity, educational level, marital status, income to poverty ratio, body mass index (BMI), risk behaviors (smoking and alcohol use), medical conditions (diabetes and hypertension), oral health (periodontal disease, tooth count, and edentulism), and use of prescription medications within the past 30 days (antibiotics, antilipidemics, and for gastroesophageal reflux disease). eAppendix 2 in Supplement 1 provides variable definitions.

Numerous bacterial genera were associated with particular covariates, with some genera associated with multiple covariates, at a Bonferroni-corrected *P* < .0002 (Figure 4). For example, prevalence of *Alloscardovia* and *Lachnospiraceae*, separately, was associated with age, race and ethnicity, smoking, alcohol use, and periodontal disease. Similarly, relative abundance of *Castellaniella* and *Phascolarctobacterium*, separately, was associated with age, race and ethnicity, periodontal disease, alcohol use, and BMI. The genera associated with each covariate generally differed for prevalence and relative abundance except for periodontal disease, with which both measures were associated with mostly the same genera (eFigure 6 in Supplement 1). Similar to α diversity, prevalence and relative abundance of several genera were nonlinearly associated with age, with varying age peaks (Figure 4; eFigure 7 in Supplement 1).

Prevalence of *Desulfomicrobium* was 6.54 (95% CI, 4.37-9.80), 5.45 (95% CI, 3.48-8.54), 4.93 (95% CI, 3.06-7.96), and 5.59 (95% CI, 3.30-9.49) times higher for individuals of Mexican American, other Hispanic, Black, and other races and ethnicities, respectively, compared with White individuals (eTable 15 in Supplement 2). Prevalence of *Metascardovia* was 12.85 (95% CI, 6.06-27.24) times higher in current smokers vs never smokers. Current smoking was also associated with increased prevalence of several genera in the *Bifidobacteriaceae* family (*Metascardovia, Scardovia, Parascardovia, Aeriscardovia,* and *Alloscardovia*). In contrast, prevalence of *Neisseria* was 0.32 (95% CI, 0.23-0.45) times higher in current smokers than never smokers. Prevalence of *Castellaniella* increased with greater alcohol use and was 4.77 (95% CI, 2.06-11.01) times higher among heavy drinkers vs never drinkers. Robust associations were also observed for relative abundance by race and ethnicity (*Murdochiella* abundance: Black vs White: ARR, 15.01 [95% CI, 5.98-37.70]; Mexican American vs White: ARR, 7.71 [95% CI, 3.22-18.43]; other Hispanic vs White: ARR, 4.63 [95% CI, 1.57-13.63]; and other races and ethnicities vs White: ARR, 18.24 [95% CI, 5.80-57.35]), smoking (*Metascardovia* abundance: 14.30 [95% CI, 3.49-58.68] times higher in current vs never smokers), and BMI (*Coprobacter* abundance: 12.00 [95% CI, 3.01-47.84] times greater among adults with severe obesity vs normal weight) (eTable 16 in Supplement 2).

Increasing severity of periodontal disease was generally associated with increased prevalence and increased relative abundance of several genera. Despite the lack of resolution in our results at the species level, both prevalence and relative abundance of red complex bacterial genera (*Porphyromonas, Tannerella,* and *Treponema*) were significantly higher among participants with severe periodontitis vs those with no periodontal disease. For example, the AOR for the presence of *Porphyromonas* was 5.21 (95% CI, 1.74-15.59) times higher for individuals with severe vs no periodontal disease (eTable 15 in Supplement 2). Similarly, the ARR for the relative abundance of *Tannerella* was 2.99 (95% CI, 2.19-4.09) times higher for individuals with severe vs no periodontal disease (eTable 16 in Supplement 2).

## Discussion

This study presents a contemporary reference standard for the oral bacterial microbiome of the US adult population aged 18 to 69 years from 2009 to 2012 (representing 202 314 000 individuals) as measured by 16S rRNA gene sequencing of oral rinse DNA. The oral microbiome is a complex ecosystem encompassing 37 phyla and over 1000 genera. Yet, a limited set of genera (*Veillonella, Streptococcus, Prevotella 7, Rothia, Actinomyces,* and *Gemella)* were observed in nearly all adults and accounted for a majority of the genus-level relative abundance (65.7%), indicating the existence of a limited universal oral microbiome in US adults.^2,12,13^ A different set of genera (*Aggregatibacter, Lactococcus,* and *Haemophilus*) were associated with a high proportion of variability in 1 measure of oral microbiome β diversity, indicating the existence of key genera that potentially affect oral microbiome diversity across individuals. A wide range of demographic, behavioral, and metabolic or clinical covariates (age, race and ethnicity, smoking, alcohol use, and periodontal disease) were consistently associated with multiple oral microbiome metrics (α diversity, β diversity, and genus-level prevalence and relative abundance), underscoring the potential modifiability of the oral microbiome.

Previous studies characterizing the oral microbiome have used convenience samples or individuals with specific health conditions. The Human Microbiome Project (HMP) established a community structure for the oral cavity across 7 oral sites, including saliva from healthy individuals with no serious oral diseases. In the HMP, the genera with the highest relative abundance in saliva were *Prevotella*, *Veillonella*, *Streptococcus*, *Fusobacterium*, an unclassified *Pasteurellaceae*, *Neisseria*, and *Porphyromonas*, making up 60.7% of the mean genus-level relative abundance. In the buccal mucosa, the top genera were *Streptococcus*, *Gemella*, an unclassified *Pasteurellaceae*, *Veillonella*, *Haemophilus*, and *Neisseria,* making up 76.1% of the total.^33^ Of the top 6 genera (from oral rinses) in the present study, 4 were highly abundant in saliva or the buccal mucosa in the HMP. The observed differences could be due to the different collection methods since oral rinse samples have been observed to have greater α diversity than samples from other oral sites and have microbial communities most similar to those in saliva.^34^ It is also possible that the differences in these data compared with the HMP are due to the nonrepresentative nature of the HMP sample or varying laboratory handling or bioinformatic processing of data.^35^

Some of the α diversity, β diversity, and genus-level associations we found have previously been noted in the literature and may have plausible biological explanations.^36,37^ Such observations include enrichment of *Actinobacteria* among current smokers (particularly several genera in the *Bifidobacteriaceae* family)^36^ and red complex bacteria in individuals with severe periodontal disease.^37^ The lower richness in edentulous individuals suggests the important role of teeth in creating diverse niches in the oral environment. Nonetheless, several of our key findings remain unexplained. The nonlinear associations of age with oral microbiome α diversity (peaking at age 30 years) and prevalence and relative abundance of several genera (varying age peaks) have not been previously reported. The numerous associations of race and ethnicity with α and β diversity metrics and genus-level prevalence and relative abundance, although previously reported,^2,11^ were not explained by the socioeconomic, behavioral, anthropometric, metabolic, and clinical factors we included for statistical adjustment.^14,38^ Additional hypotheses and research are needed to identify potential biological or alternative explanations. For example, the nonlinear association with age could arise from biological aging or reflect birth cohort specific patterns. However, we could not evaluate these birth cohort specific patterns because the narrow calendar years in this study made age equivalent to birth cohort. Similarly, associations with race and ethnicity could reflect multifactorial social determinants of health, such as early life exposures or differential access to health services,^38^ that were not considered herein.

### Strengths and Limitations

The strengths of this study include the population-representative nature of the data, large sample size, and the comprehensive array of demographic, socioeconomic, and behavioral data. Additionally, we used uniform, high-quality data and unindicated (ie, assessment independent of health status) health examinations.

This study has several limitations. Our evaluation was restricted to 16S rRNA gene sequencing because metagenomic sequencing was precluded by NCHS policy. Thus, the data resolution was through the genus level, and we lacked species differentiation and imputed bacterial functionality, which metagenomic sequencing would have permitted. In addition, due to the short sequencing length, we were unable to include additional error correction by merging the paired-end reads. Furthermore, we cannot exclude the potential of residual confounding from diet, oral hygiene, or other factors.

## Conclusions

In this cross-sectional study of the oral microbiome of adults who participated in 2 cycles of NHANES, we characterized the composition, diversity, and correlates of the oral microbiome (colloquially, the mouth of America). The NCHS made all the NHANES results available to the research community. This comprehensive characterization, along with the anticipated use of the data by the scientific community, portends a deeper understanding of the role of the oral microbiome in human health. It also provides a contemporary reference standard for future studies.

## Notes

Supplement 1. **eAppendix 1.** Characteristics of NHANES 2009-2012 Participants Aged 18-69 Years Old With or Without Oral Microbiome Data **eAppendix 2.** NHANES Variable Definitions for Analysis **eFigure 1.** Relative Abundance of Major Phyla in Quality Control Samples Across Sequencing Plates and Batches **eFigure 2.** Relative Abundance of Major Genera in Quality Control Samples Across Sequencing Plates and Batches **eFigure 3.** Scree Plot of Principal Coordinate Analysis (PCoA) Vectors for Bray-Curtis, Unweighted UniFrac, and Weighted UniFrac Beta Diversity Matrices **eFigure 4.** Associations of Covariates With Alpha Diversity Metrics Including Observed ASVs (A), Faith’s Phylogenetic Diversity (B), the Simpson Index (C), and the Shannon-Weiner Index (D) **eFigure 5.** Proportion of Variability in the Unweighted UniFrac Beta Diversity Matrix From Prevalence of Each Genus (A) and From Relative Abundance of Key Genera (B) and in the Weighted UniFrac (C) and Bray-Curtis (D) Beta Diversity Matrices From Relative Abundance of Key Genera **eFigure 6.** Heatmap of Associations Between Covariates and the Genus-Level Presence and Relative Abundance **eFigure 7.** Model-Predicted Genus-Specific Sequence Counts by Age (18-69 Years) for Genera With Association of Genus-Specific Relative Abundance With Age
eReference

Supplement 2. **eTable 1.** Taxonomy Identifiers From SILVA v123 **eTable 2.** Phylum-Level Oral Microbiome Prevalence Estimates, NHANES 2009-2012 **eTable 3.** Class-Level Oral Microbiome Prevalence Estimates, NHANES 2009-2012 **eTable 4.** Order-Level Oral Microbiome Prevalence Estimates, NHANES 2009-2012 **eTable 5.** Family-Level Oral Microbiome Prevalence Estimates, NHANES 2009-2012 **eTable 6.** Genus-Level Oral Microbiome Prevalence Estimates, NHANES 2009-2012 **eTable 7.** Weighted Pearson Correlation Coefficients and *P*-values From the Phylum-Level Relative Abundances, NHANES 2009-2012 **eTable 8.** Weighted Pearson Correlation Coefficients and *P*-values From the Class-Level Relative Abundances, NHANES 2009-2012 **eTable 9.** Weighted Pearson Correlation Coefficients and *P*-values From the Order-Level Relative Abundances, NHANES 2009-2012 **eTable 10.** Weighted Pearson Correlation Coefficients and *P*-values From the Family-Level Relative Abundances, NHANES 2009-2012 **eTable 11.** Weighted Pearson Correlation Coefficients and *P*-values From the Genus-Level Relative Abundances, NHANES 2009-2012 **eTable 12.** Associations Between Covariates and the First 100 Principal Coordinate Analysis (PCoA) Vectors From the Bray-Curtis Dissimilarity Matrix Mutually Adjusted for All Independent Variables **eTable 13.** Associations Between Covariates and the First 100 Principal Coordinate Analysis (PCoA) Vectors From the Unweighted UniFrac Matrix Mutually Adjusted for All Independent Variables **eTable 14.** Associations Between Covariates and the First 100 Principal Coordinate Analysis (PCoA) Vectors From the Weighted UniFrac Matrix Mutually Adjusted for All Independent Variables **eTable 15.** Associations Between Covariates and Genus-Level Presence From Logistic Regression Models Mutually Adjusted for All Independent Variables **eTable 16.** Associations Between Covariates and Genus-Level Relative Abundance From Poisson Regression Models Mutually Adjusted for All Independent Variables

Supplement 3.
Data Sharing Statement
