# Supplementary material for: Oral Microbiome Profile of the US Population
Source: JAMA Netw Open. 2025 May 5;8(5):e258283. doi: 10.1001/jamanetworkopen.2025.8283 (PMC12053784; doi:10.1001/jamanetworkopen.2025.8283)
Supplement: Supplement 1. — eAppendix 1. Characteristics of NHANES 2009-2012 Participants Aged 18-69 Years Old With or Without Oral Microbiome Data eAppendix 2. NHANES Variable Definitions for Analysis eFigure 1. Relative Abundance of Major Phyla in Quality Control Samples Across Sequencing Plates and Batches eFigure 2. Relative Abundance of Major Genera in Quality Control Samples Across Sequencing Plates and Batches eFigure 3. Scree Plot of Principal Coordinate Analysis (PCoA) Vectors for Bray-Curtis, Unweighted UniFrac, and Weighted UniFrac Beta Diversity Matrices eFigure 4. Associations of Covariates With Alpha Diversity Metrics Including Observed ASVs (A), Faith’s Phylogenetic Diversity (B), the Simpson Index (C), and the Shannon-Weiner Index (D) eFigure 5. Proportion of Variability in the Unweighted UniFrac Beta Diversity Matrix From Prevalence of Each Genus (A) and From Relative Abundance of Key Genera (B) and in the Weighted UniFrac (C) and Bray-Curtis (D) Beta Diversity Matrices From Relative Abundance of Key Genera eFigure 6. Heatmap of Associations Between Covariates and the Genus-Level Presence and Relative Abundance eFigure 7. Model-Predicted Genus-Specific Sequence Counts by Age (18-69 Years) for Genera With Association of Genus-Specific Relative Abundance With Age eReference [file jamanetwopen-e258283-s001.pdf]

Supplemental Online Content

Chaturvedi AK, Vogtmann E, Shi J, et al. Oral microbiome profile of the US population. *JAMA Netw Open*. 2025;8(5):e258283. doi:10.1001/jamanetworkopen.2025.8283

eAppendix 1. Characteristics of NHANES 2009-2012 Participants Aged 18-69 Years Old With or Without Oral Microbiome Data
eAppendix 2. NHANES Variable Definitions for Analysis
eFigure 1. Relative Abundance of Major Phyla in Quality Control Samples Across Sequencing Plates and Batches
eFigure 2. Relative Abundance of Major Genera in Quality Control Samples Across Sequencing Plates and Batches
eFigure 3. Scree Plot of Principal Coordinate Analysis (PCoA) Vectors for Bray-Curtis, Unweighted UniFrac, and Weighted UniFrac Beta Diversity Matrices
eFigure 4. Associations of Covariates With Alpha Diversity Metrics Including Observed ASVs (A), Faith's Phylogenetic Diversity (B), the Simpson Index (C), and the Shannon-Weiner Index (D)
eFigure 5. Proportion of Variability in the Unweighted UniFrac Beta Diversity Matrix From Prevalence of Each Genus (A) and From Relative Abundance of Key Genera (B) and in the Weighted UniFrac (C) and Bray-Curtis (D) Beta Diversity Matrices From Relative Abundance of Key Genera
eFigure 6. Heatmap of Associations Between Covariates and the Genus-Level Presence and Relative Abundance
eFigure 7. Model-Predicted Genus-Specific Sequence Counts by Age (18-69 Years) for Genera With Association of Genus-Specific Relative Abundance With Age
eReference

This supplemental material has been provided by the authors to give readers additional information about their work.

© 2025 Chaturvedi AK. *JAMA Network Open*

eAppendix 1. Characteristics of NHANES 2009-2012 participants aged 18-69 years old with or without oral microbiome data

	**Without oral microbiome data, ages 18-69**		**With oral microbiome data, ages 18-69**	
	**N = 1861**		**N = 8237**	
	**N**	**(Weighted %) [a]**	**N**	**(Weighted %) [a]**
Age groups (years)				
18-29	409	21.6	2164	25.1
30-39	362	18.9	1573	19.6
40-49	368	20.6	1594	21.3
50-59	368	22.9	1445	20.0
60-69	354	16.1	1461	14.0
Sex				
Male	1014	54.5	4127	50.0
Female	847	45.4	4110	50.0
Self-reported race and ethnicity				
Mexican American	237	6.8	1387	9.5
Other Hispanic	239	6.8	853	6.0
Non-Hispanic White	790	68.6	3064	64.1
Non-Hispanic Black	392	10.7	1961	12.4
Other races and ethnicities	203	7.1	972	8.0
Education				
Less than high school	490	1.1	2053	17.0
Completed high school/GED	422	22.5	1831	21.1
More than high school	949	60.4	4353	61.9
Marital status				
Never married	473	23.2	2267	24.4
Married/living with partner	1029	60.3	4560	60.7

© 2025 Chaturvedi AK. *JAMA Network Open*

Divorced/widowed/separated	359	16.5	1410	14.9
Income-to-poverty ratio				
<1 (below poverty level)	434	15.0	1985	16.7
1-1.999	409	17.2	1975	18.4
2-2.999	237	13.9	947	12.7
≥3	570	46.3	2641	45.4
Missing	211	7.6	689	6.7
Body mass index (kg/m^2) categories				
<18.5 (Underweight)	47	2.4	150	1.9
18.5-24.999 (Normal weight)	595	31.5	2463	30.5
25-29.999 (Overweight)	563	32.8	2614	32.4
30-34.999 (Obesity)	377	18.9	1653	20.1
≥35 (Severe obesity)	279	14.3	1357	15.1
Cigarette smoking history				
Never	1058	56.9	4051	56.2
Former	311	18.4	1408	18.2
Current	492	24.7	2178	25.5
Alcohol consumption				
Never drinker	213	7.9	1032	9.3
Drinks 0 drinks/week in past 12 months	191	8.9	1124	12.3
Drinks >0-<1 drink/week in past 12 months	382	21.5	2351	28.0
Drinks 1-<8 drinks/week in past 12 months	387	24.9	1931	27.7
Drinks 8-<14 drinks/week in past 12 months	77	5.0	527	7.9
Drinks ≥14 drinks/week in past 12 months	102	6.2	559	7.5
Missing	509	25.6	713	7.4
Diabetes				
No	1624	89.6	7210	90.1
Yes	237	10.4	1117	9.9

© 2025 Chaturvedi AK. *JAMA Network Open*

Hypertension				
No	1063	59.1	1861	58.2
Yes	798	40.9	4621	41.8
Periodontal disease/edentulism				
None	365	25.3	2551	37.8
Mild	57	2.7	365	4.3
Moderate	252	13.0	1720	18.4
Severe	84	4.1	676	5.8
Not eligible (<30 years old)	409	21.6	2164	25.1
Edentulous	47	1.6	271	2.5
Missing	647	31.8	490	6.0
Antibiotic use				
No	1796	96.3	7949	96.2
Yes	65	3.7	288	3.8
Anti-gastroesophageal reflux medication				
No	1684	89.5	1861	91.6
Yes	177	10.5	7537	8.5
Anti-hyperlipidemic medication				
No	1618	86.6	7158	86.8
Yes	243	13.4	1079	13.2
Inhaled respiratory medication				
No	1851	99.6	8186	99.4
Yes	10	0.4	51	0.6

[a] MEC weights were used to estimate the weighted percentages.

© 2025 Chaturvedi AK. *JAMA Network Open*

eAppendix 2. NHANES variable definitions for analysis

Variable	Source/creation
**Age**, modeled as restricted 5-knot cubic regression splines	Based on self-reported age at interview
**Sex**	Based on self-reported sex at interview
Male	
Female	
**Self-reported race and ethnicity**	Based on self-reported race and ethnicity at interview. No collapsing of groups was conducted.
Mexican American	
Other Hispanic	
Non-Hispanic Black	
Non-Hispanic White	
Non-Hispanic other race including multiracial	
**Education**	Self-reported level of highest education. Missing values were assigned the mode—more than high school.
Less than high school	
Completed high school/GED	
More than high school	
**Marital status**	Self-reported marital status. Missing values were assigned the mode—married/living with partner.
Never married	
Married/living with partner	
Divorced/widowed/separated	
**Income-to-poverty ratio**	Self-reported family income divided by the federal poverty level. Missing values were coded as a separate category.
<1 (below poverty level)	
1-1.999	
2-2.999	

© 2025 Chaturvedi AK. *JAMA Network Open*

≥3	
Missing	
**Body mass index (kg/m^2) categories**	Based on measured height and weight. Missing values were assigned the mode—normal weight category.
<18.5 (Underweight)	
18.5-24.999 (Normal weight)	
25-29.999 (Overweight)	
30-34.999 (Obesity)	
≥35 (Severe obesity)	
**Cigarette smoking history**	Self-reported smoking status, does not consider serum cotinine levels. Missing values were assigned the mode—never smoker.
Never	
Former	
Current	
**Alcohol consumption**	Self-reported alcohol intake over the past 12 months, expressed as drinks per week. Missing values were coded as a separate category.
Never drinker	
Drinks 0 drinks/week in past 12 months	
Drinks >0-<1 drink/week in past 12 months	
Drinks 1-<8 drinks/week in past 12 months	
Drinks 8-<14 drinks/week in past 12 months	
Drinks ≥14 drinks/week in past 12 months	
Missing	
**Diabetes**	Based on self-report of diabetes, self-report of taking insulin or other diabetes medication, measured hemoglobin A1C of >= 6.5, or measured serum glucose levels >=126. Missing values were assigned the mode—no diabetes.
No	
Yes	

© 2025 Chaturvedi AK. *JAMA Network Open*

**Hypertension**	Based on self-report of hypertension, self-report of taking hypertension medication, measured diastolic pressure >80 or measured systolic pressure >130. Missing values were assigned the mode—no hypertension.
No	
Yes	
**Periodontal disease/edentulism**	Combined variable for severity of periodontal disease and edentulism. Periodontal disease severity was based on periodontal disease examination; edentulism was based on measured count of teeth. Missing values were coded as a separate category.
None	
Mild	
Moderate	
Severe	
Not eligible (<30 years old)	
Edentulous	
Missing	
**Antibiotic use**	Based on self-reported use of medications in the past 30 days. Missing values were assigned the mode—no use.
No	
Yes	
**Anti-gastroesophageal reflux medication**	Based on self-reported use of medications in the past 30 days. Missing values were assigned the mode—no use.
No	
Yes	
**Anti-hyperlipidemic medication**	Based on self-reported use of medications in the past 30 days. Missing values were assigned the mode—no use.
No	
Yes	
**Inhaled respiratory medication**	Based on self-reported use of medications in the past 30 days. Missing values were assigned the mode—no use.
No	

© 2025 Chaturvedi AK. *JAMA Network Open*

Yes	

NOTE: Missing values were assigned to the most prevalent category (mode) when fewer than 5% of participants had missing observations. Missing values were treated as a separate category when >5% of participants had missing observations.

© 2025 Chaturvedi AK. *JAMA Network Open*

eFigure 1. Relative abundance of major phyla in quality control samples across sequencing plates and batches. AC1: Aliquot 1 from the MicroBiome Quality Control (MBQC)[1] project oral artificial community; AC2: Aliquot 2 from the MBQC project oral artificial community; RG1: Aliquot 1 from the MBQC robogut sample; RG2: Aliquot 2 from the MBQC robogut sample. The numbers in parentheses represent the number of samples of that type.

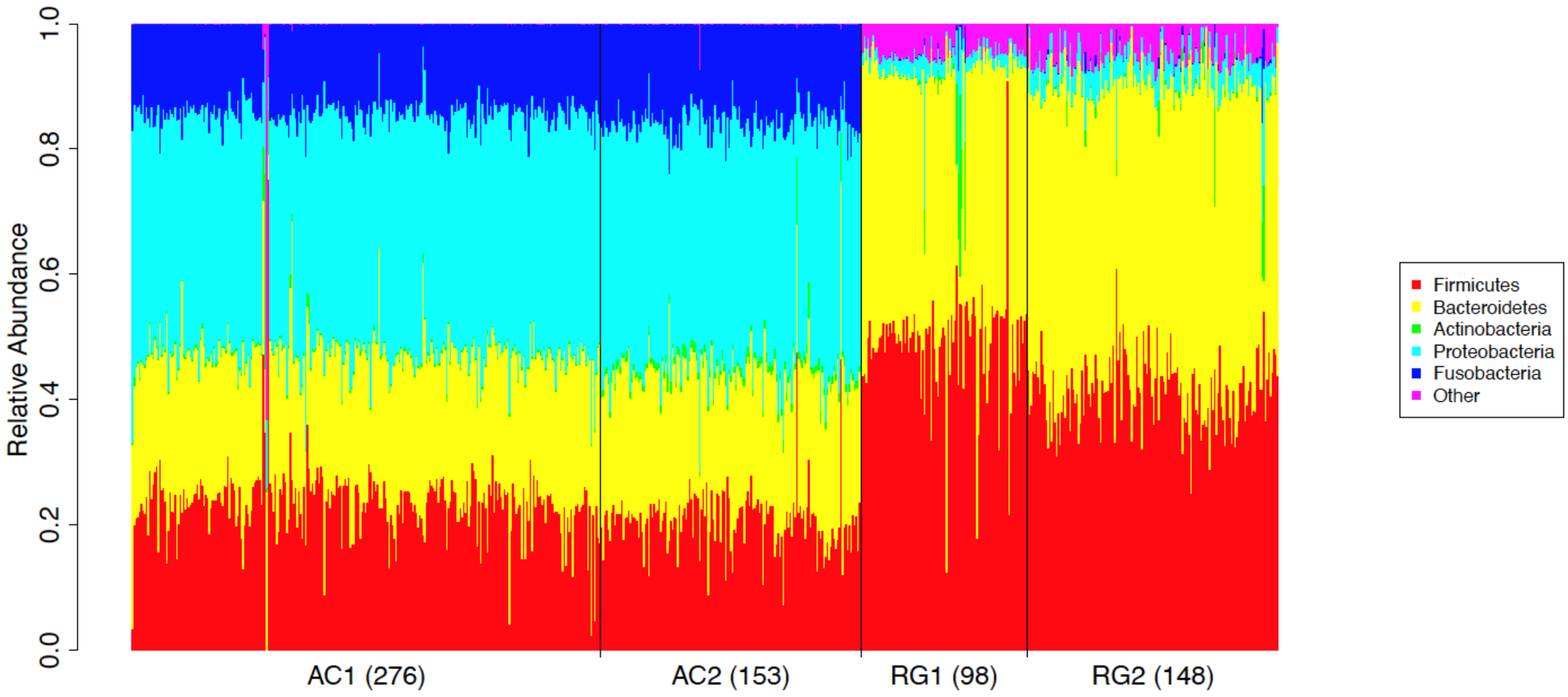

© 2025 Chaturvedi AK. *JAMA Network Open*

eFigure 2. Relative abundance of major genera in quality control samples across sequencing plates and batches. AC1: Aliquot 1 from the MicroBiome Quality Control (MBQC)[1] project oral artificial community; AC2: Aliquot 2 from the MBQC project oral artificial community; RG1: Aliquot 1 from the MBQC robogut sample; RG2: Aliquot 2 from the MBQC robogut sample. The numbers in parentheses represent the number of samples of that type.

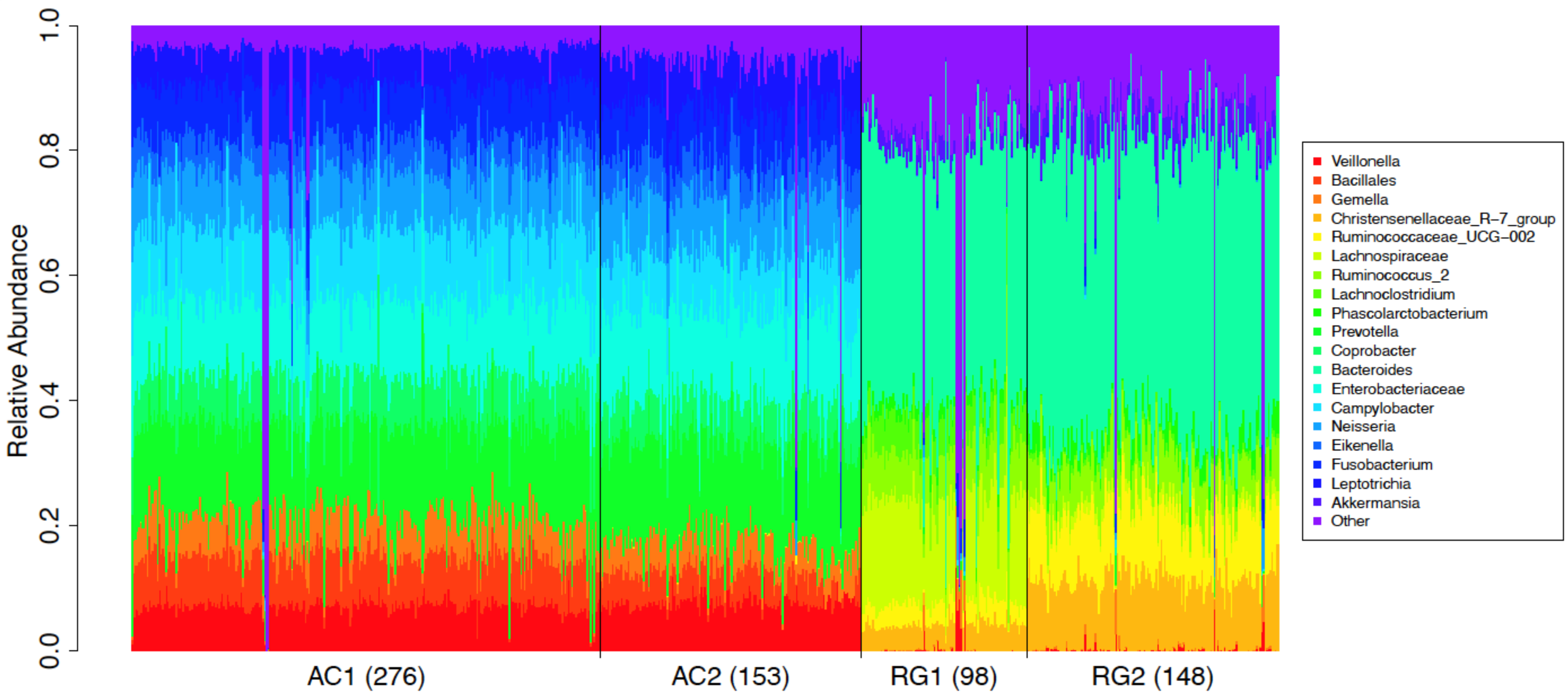

© 2025 Chaturvedi AK. *JAMA Network Open*

eFigure 3. Scree plot of principal coordinate analysis (PCoA) vectors for Bray-Curtis, unweighted UniFrac, and weighted UniFrac beta diversity matrices.

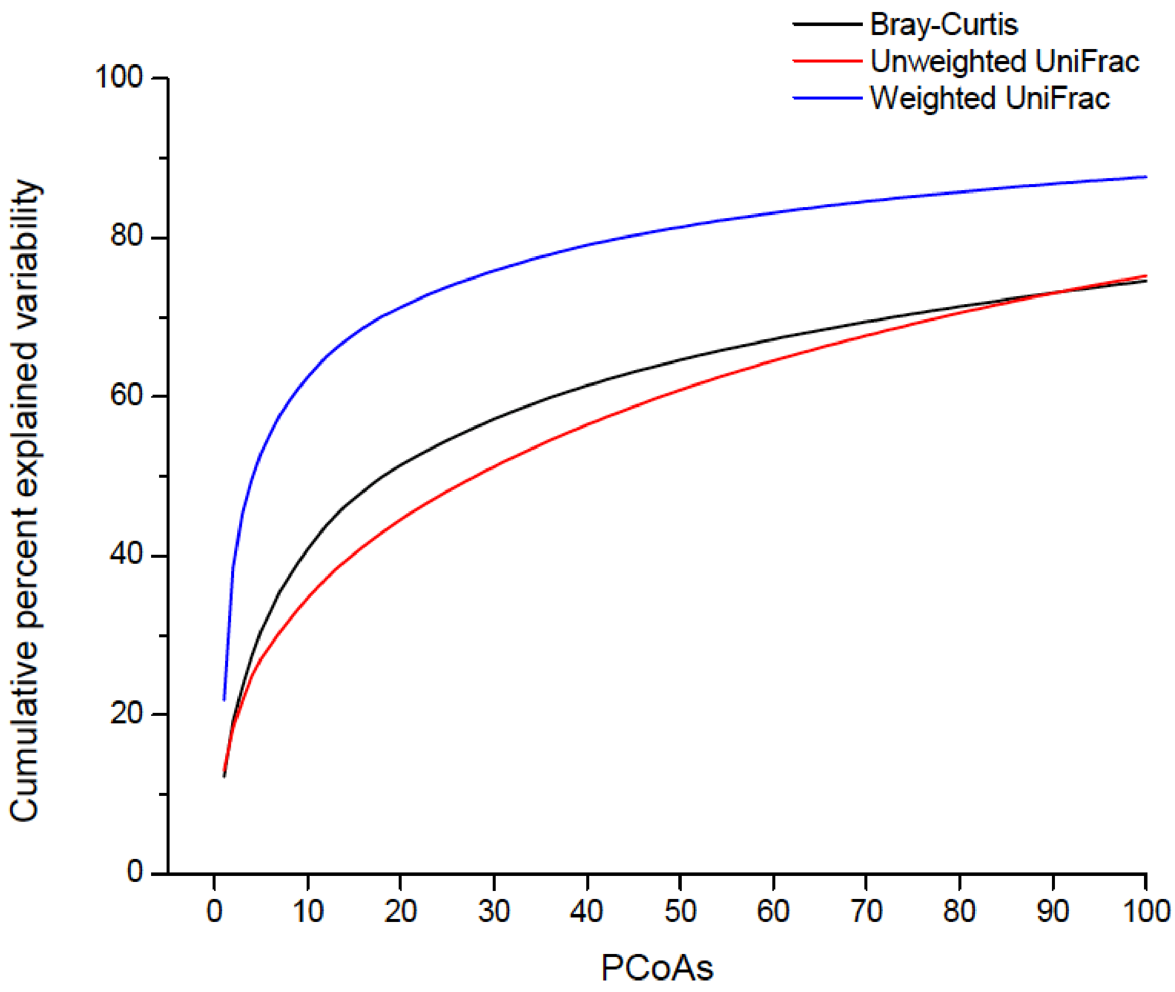

© 2025 Chaturvedi AK. *JAMA Network Open*

eFigure 4. Associations of covariates with alpha diversity metrics including observed ASVs (A), Faith's phylogenetic diversity (B), the Simpson Index (C), and the Shannon-Weiner Index (D). Predictive margins were estimated in survey-design linear regression models. For each metric the overall estimate is from an unadjusted model. All other estimates include concomitant adjustment for age (modeled as 5-knot restricted cubic splines), sex, race and ethnicity, education, marital status, income-to-poverty ratio, measured body mass index [BMI] categories, risk behaviors (smoking, alcohol use), medical conditions (diabetes and hypertension), oral health (periodontal disease, tooth count, and edentulism), and use of prescription medications within the past 30 days (antibiotics, antilipidemics, respiratory inhalants, and for gastroesophageal reflux). Race and ethnicity was self-reported and used as categorized by NHANES. [a] denotes p<0.05 for the covariate in the adjusted model.

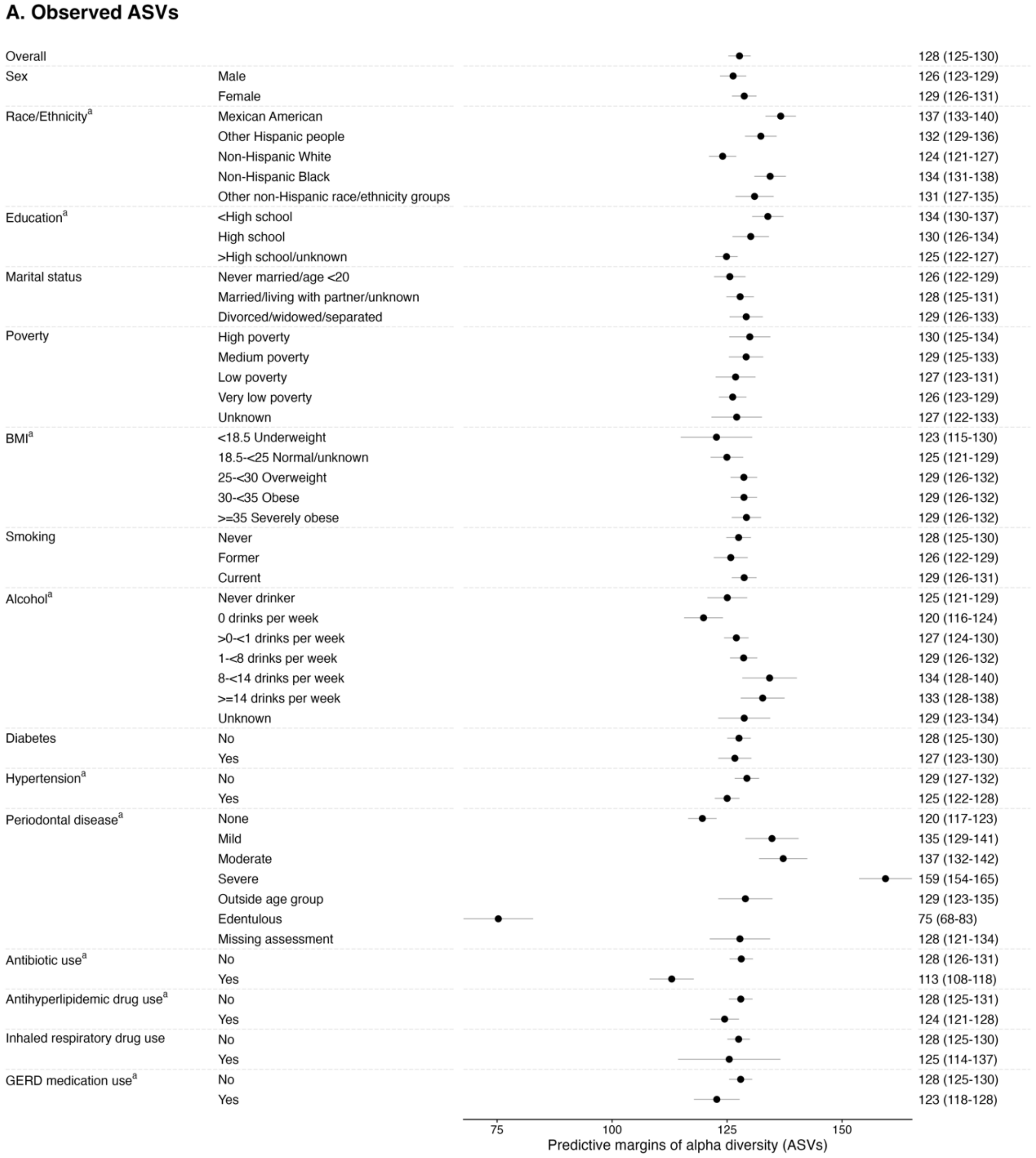

© 2025 Chaturvedi AK. *JAMA Network Open*

B. Faith's PD

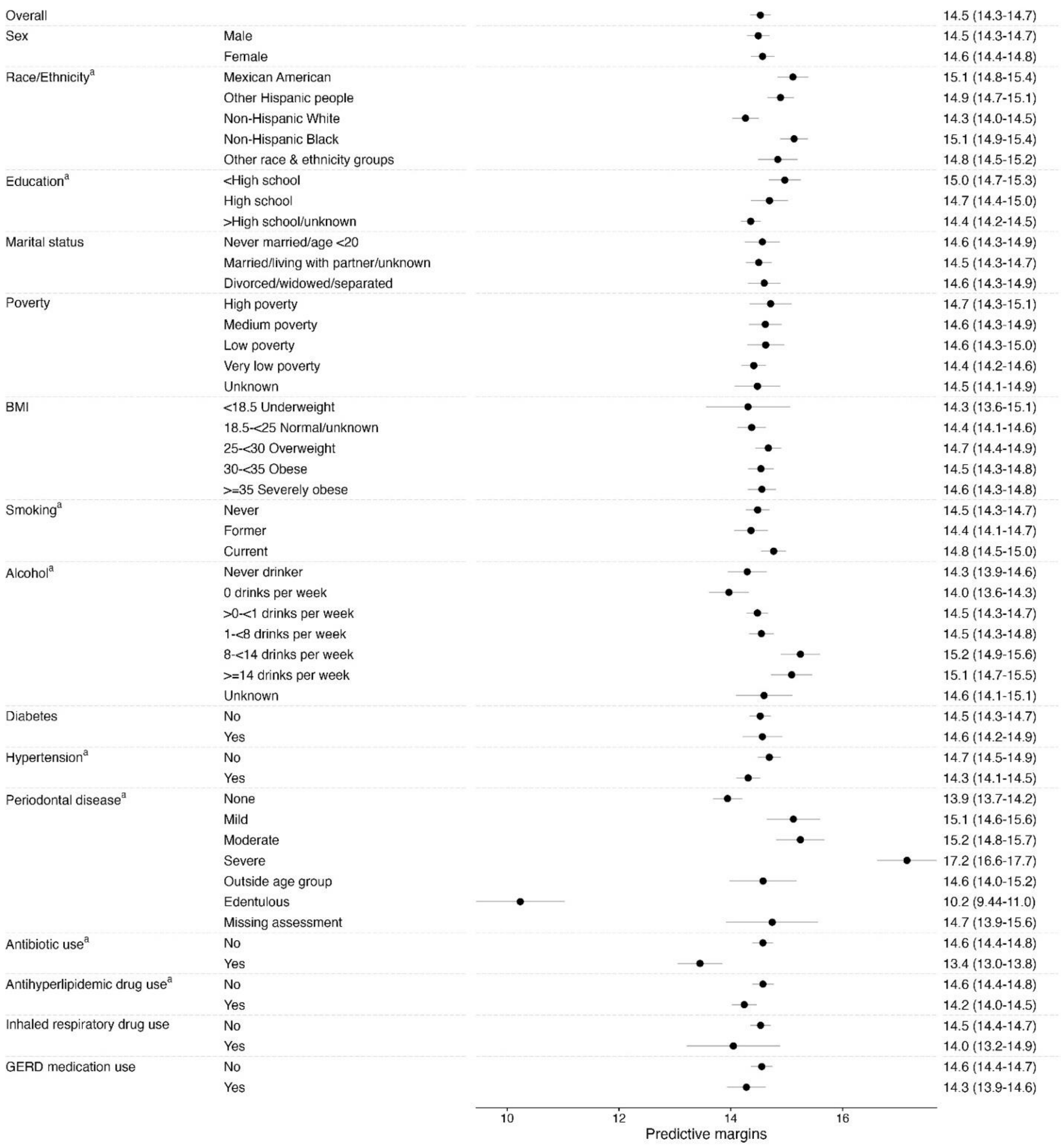

© 2025 Chaturvedi AK. *JAMA Network Open*

C. Simpson index

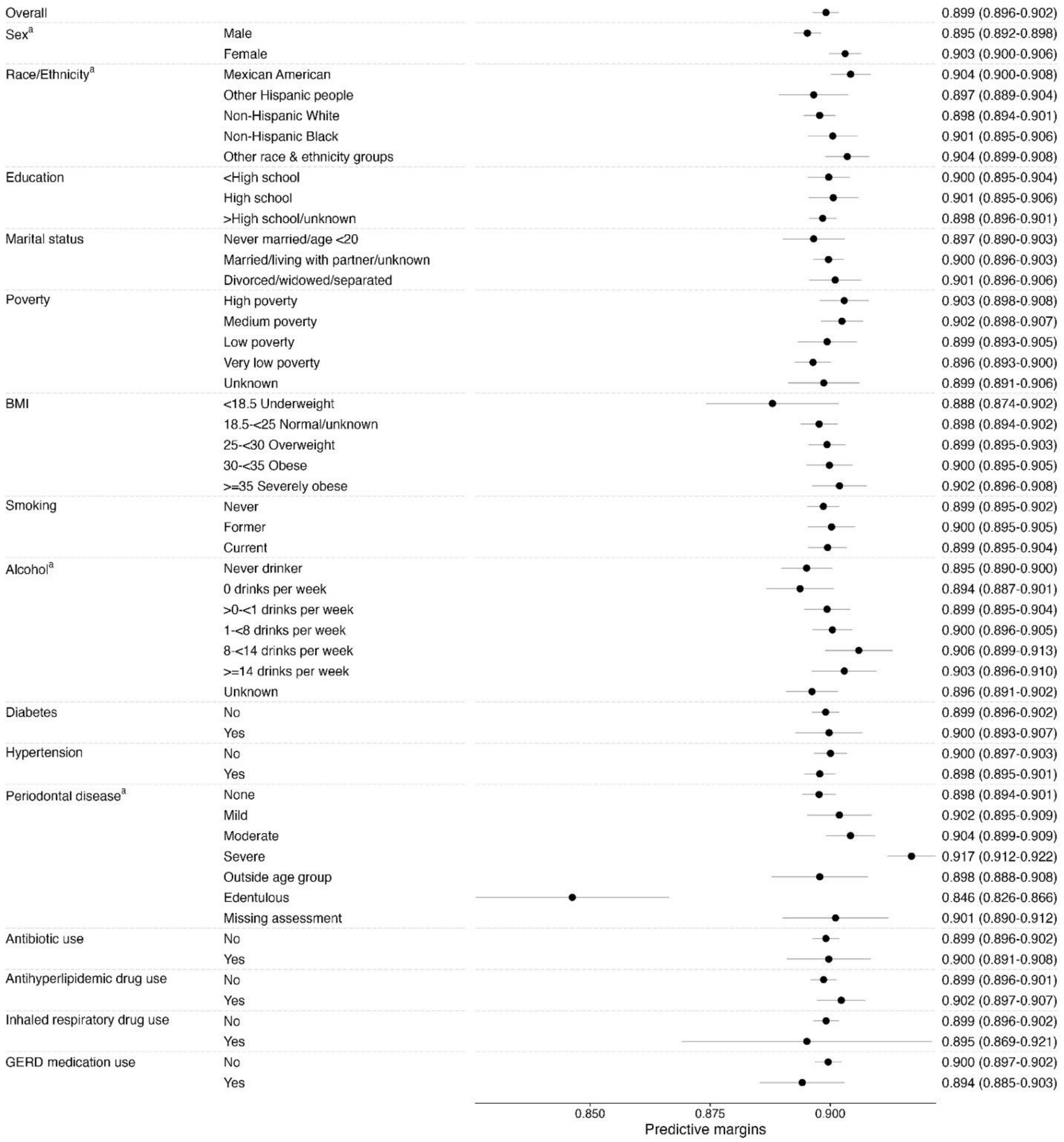

© 2025 Chaturvedi AK. *JAMA Network Open*

D. Shannon-Weiner index

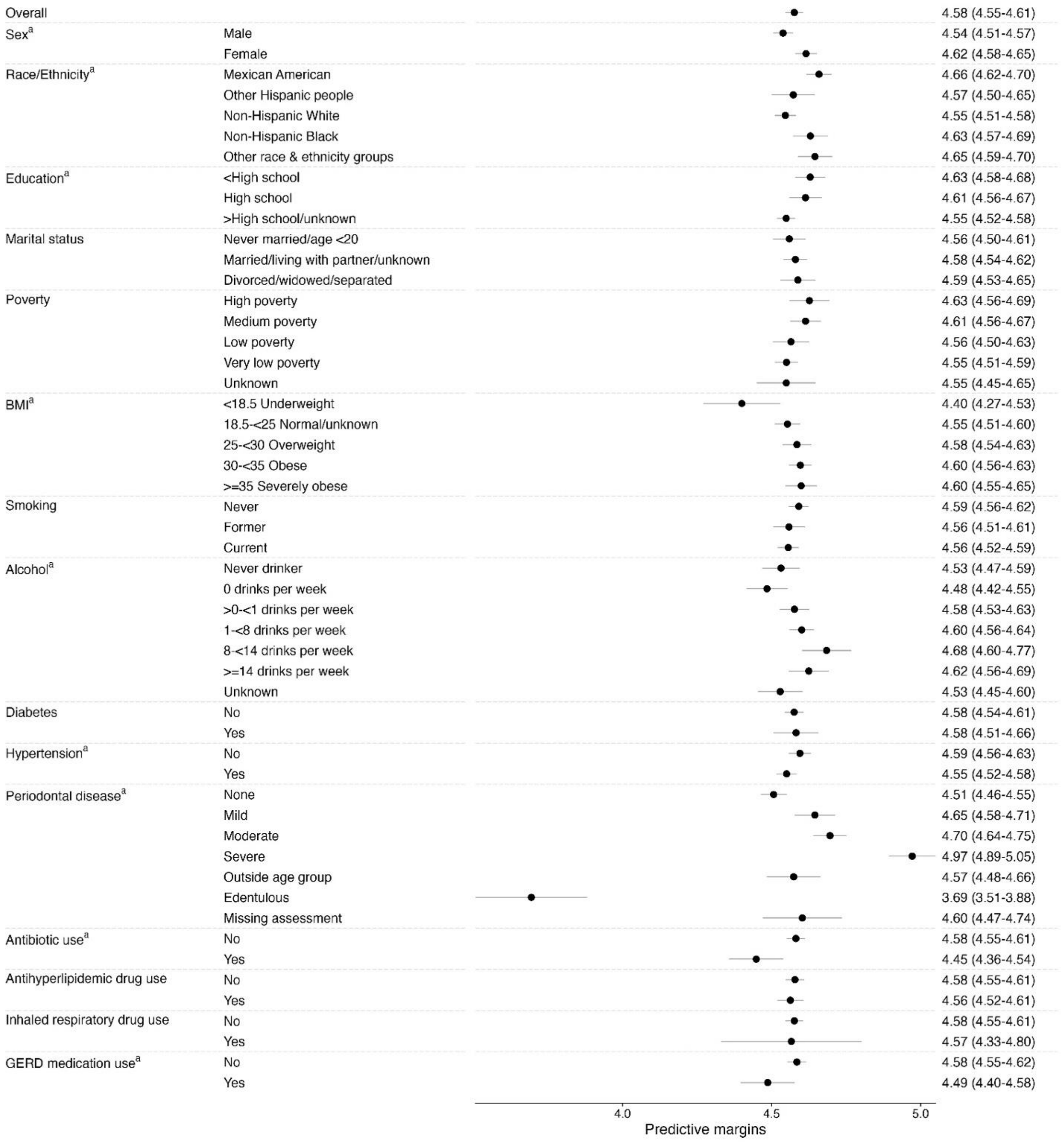

© 2025 Chaturvedi AK. *JAMA Network Open*

eFigure 5. Proportion of variability in the unweighted UniFrac beta diversity matrix from prevalence of each genus (A) and from relative abundance of key genera (B) and in the weighted UniFrac (C) and Bray-Curtis (D) beta diversity matrices from relative abundance of key genera. The fastAdonis algorithm was used for estimation and genus-specific estimates are unadjusted for covariates or correlations across prevalence or relative abundance of genera.

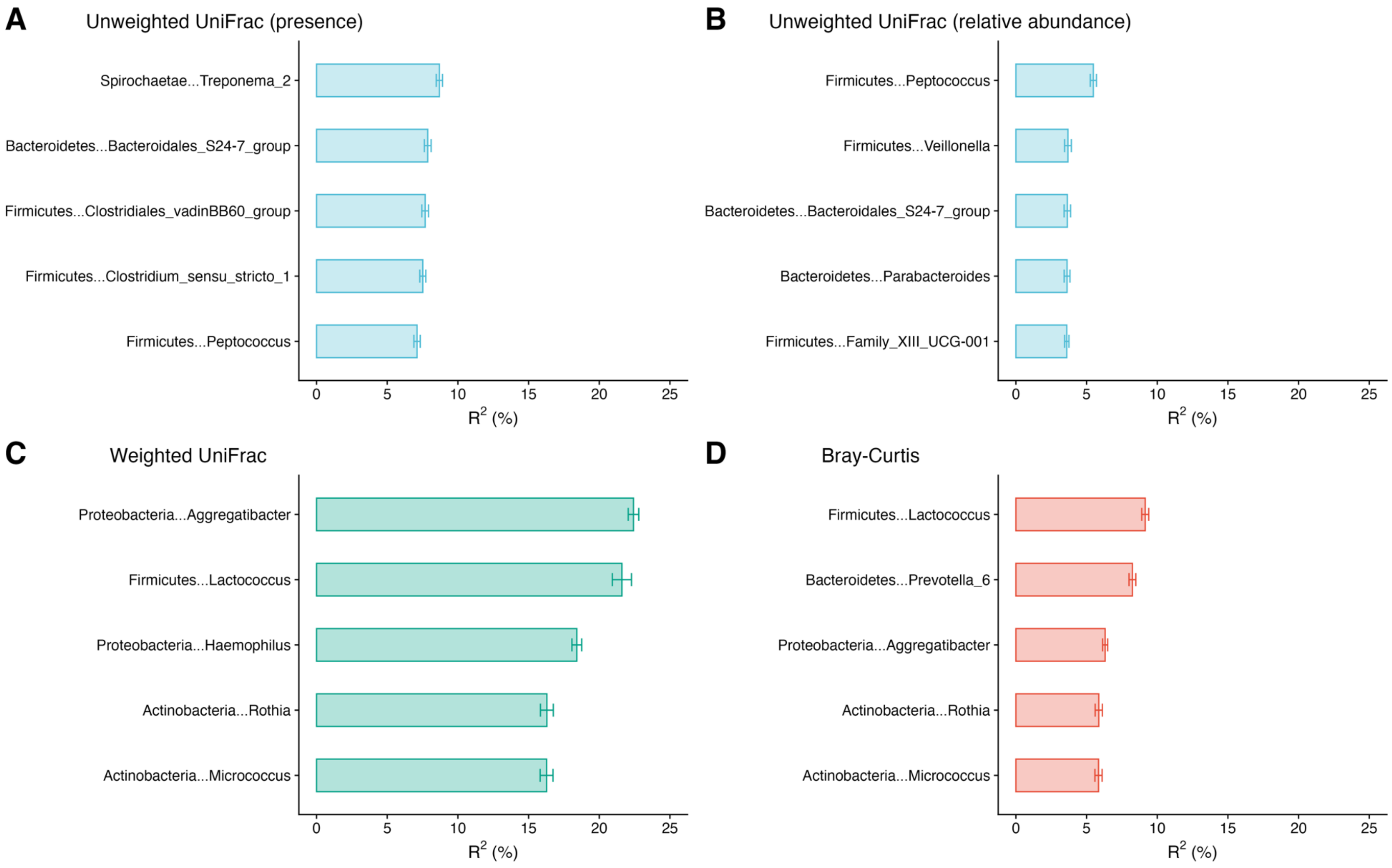

© 2025 Chaturvedi AK. *JAMA Network Open*

eFigure 6. Heatmap of associations between covariates and the genus-level presence and relative abundance. Colored rectangles indicate an association with a p-value < 0.0002 for associations with age (tan), periodontal disease (red), race/ethnicity (blue), smoking (orange), alcohol (green), and body mass index (BMI, brown). All models included concomitant adjustment for age (modeled as 5-knot restricted cubic splines), sex, race and ethnicity, education, marital status, income-to-poverty ratio, measured body mass index [BMI] categories, risk behaviors (smoking, alcohol use), medical conditions (diabetes and hypertension), oral health (periodontal disease, tooth count, and edentulism), and use of prescription medications within the past 30 days (antibiotics, antilipidemics, and for gastroesophageal reflux). Race and ethnicity was self-reported and used as categorized by NHANES.

© 2025 Chaturvedi AK. *JAMA Network Open*

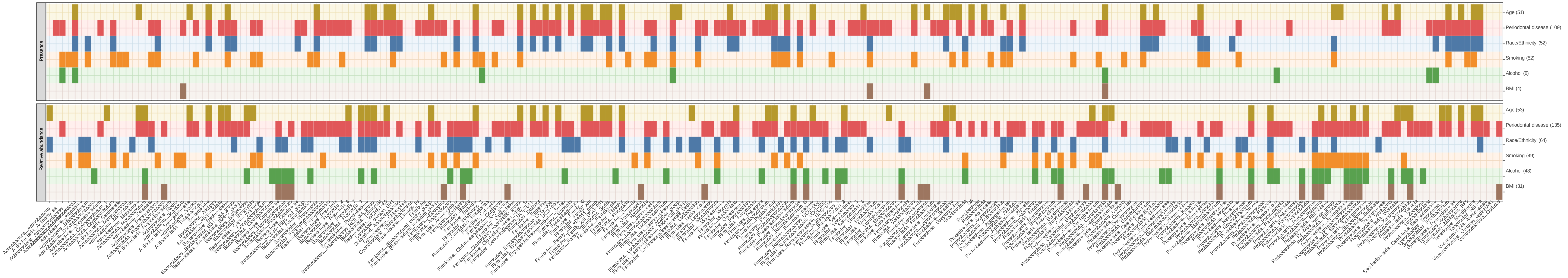
Presence
Age (51)
Periodontal disease (109)
Race/Ethnicity (52)
Smoking (52)
Alcohol (8)
BMI (4)
Relative abundance
Age (53)
Periodontal disease (135)
Race/Ethnicity (64)
Smoking (49)
Alcohol (48)
BMI (31)

eFigure 7. Model-predicted genus-specific sequence counts by age (18-69 years) for genera with association of genus-specific relative abundance with age. All models included concomitant adjustment for age (modeled as 5-knot restricted cubic splines), sex, race and ethnicity, education, marital status, income-to-poverty ratio, measured body mass index [BMI] categories, risk behaviors (smoking, alcohol use), medical conditions (diabetes and hypertension), oral health (periodontal disease, tooth count, and edentulism), and use of prescription medications within the past 30 days (antibiotics, antilipidemics, and for gastroesophageal reflux). Race and ethnicity was self-reported and used as categorized by NHANES.

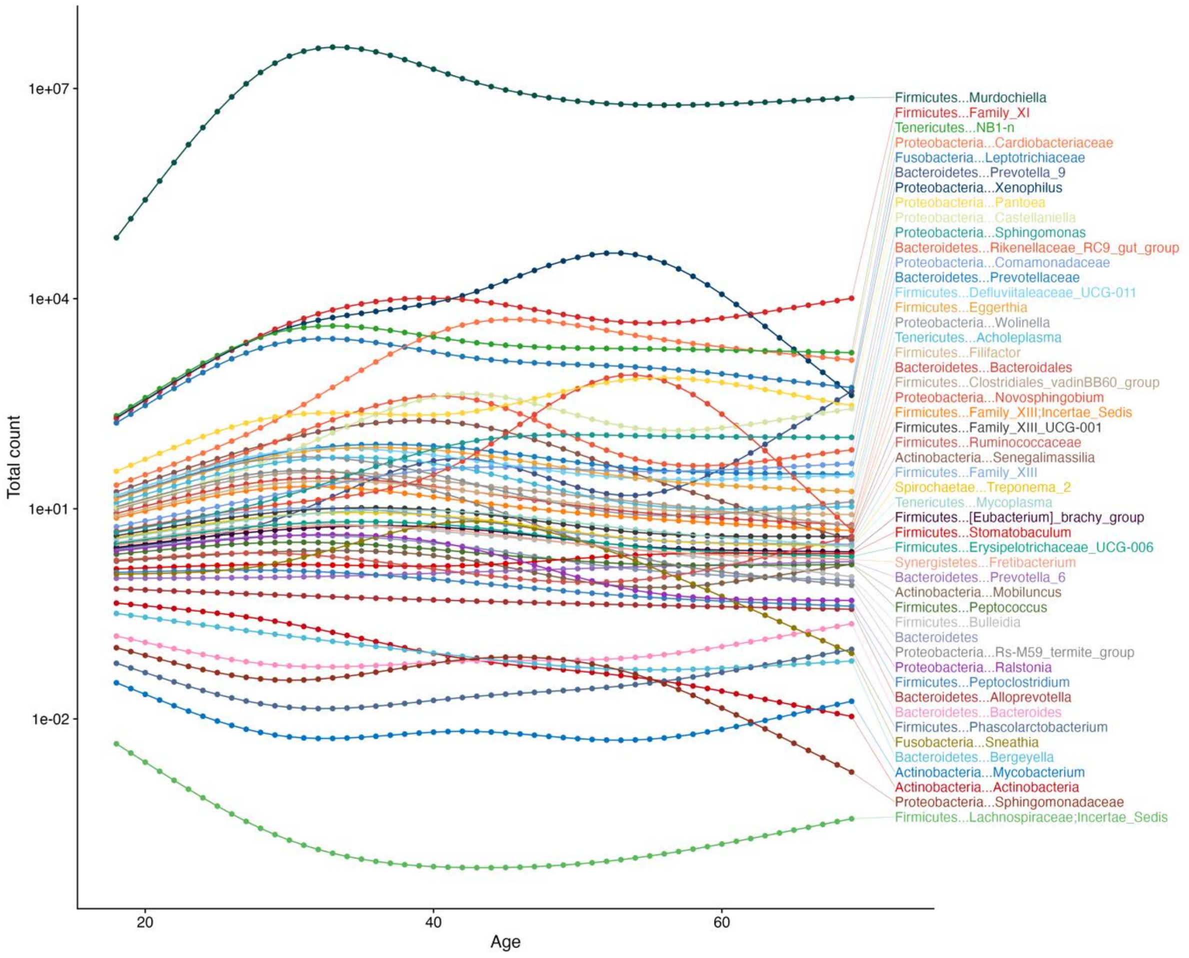

© 2025 Chaturvedi AK. *JAMA Network Open*

eReference

1. Sinha R, Abu-Ali G, Vogtmann E, et al. Assessment of variation in microbial community amplicon sequencing by the Microbiome Quality Control (MBQC) project consortium. *Nat Biotechnol*. Nov 2017;35(11):1077-1086. doi:10.1038/nbt.3981

© 2025 Chaturvedi AK. *JAMA Network Open*
