# Supplementary material for: Oral Microbiome Profile of the US Population
Source: JAMA Netw Open. 2025 May 5;8(5):e258283. doi: 10.1001/jamanetworkopen.2025.8283 (PMC12053784; doi:10.1001/jamanetworkopen.2025.8283)
Supplement: Supplement 3. — Data Sharing Statement [file jamanetwopen-e258283-s003.pdf]

Data Sharing Statement

Chaturvedi. Oral Microbiome Profile of the US Population. *JAMA Netw Open*. Published May 05, 2025. doi:10.1001/jamanetworkopen.2025.8283

Data
Data available: Yes
Data types: Deidentified participant data, Data dictionary
How to access data: https://wwwn.cdc.gov/Nchs/Nhanes/omp/
When available: beginning date: 11-27-2024

Supporting Documents
Document types: None

Additional Information
Who can access the data: NHANES determines eligibility to use the data. These data are available for public use now.
Types of analyses: NHANES will determine analytic parameters if any of the non-public data are requested.
Mechanisms of data availability: NHANES has the public use data available for download now.
